# *MYC* gene amplification is a rare event in atypical fibroxanthoma and pleomorphic dermal sarcoma

**DOI:** 10.18632/oncotarget.24997

**Published:** 2018-04-20

**Authors:** Timo Gaiser, Daniela Hirsch, Azadeh Orouji, Marisa Bach, Peter Kind, Doris Helbig, Alexander Quaas, Jochen Utikal, Alexander Marx, Maria Rita Gaiser

**Affiliations:** ^1^ Institute of Pathology, University Medical Center Mannheim, Ruprecht-Karl University of Heidelberg, Mannheim, Germany; ^2^ Department of Dermatology, Venereology and Allergology, University Medical Center Mannheim, Ruprecht-Karl University of Heidelberg, Mannheim, Germany; ^3^ Skin Cancer Unit, German Cancer Research Center (DKFZ), Heidelberg, Germany; ^4^ Dermatohistological Laboratory Professor Kind, Offenbach, Germany; ^5^ Department of Dermatology, University Hospital Cologne, Cologne, Germany; ^6^ Institute of Pathology, University Hospital Cologne, Cologne, Germany

**Keywords:** AFX, amplification, FISH, MYC, PDS

## Abstract

Atypical fibroxanthoma (AFX) and pleomorphic dermal sarcoma (PDS) are rare malignancies typically occurring in elderly patients and predominantly located in skin regions exposed to UV-light. Thus, a role of UV-radiation-induced damage for AFX and PDS tumorigenesis has been postulated.

*MYC* gene amplification has been demonstrated as a distinctive feature of radiation-induced angiosarcoma. In order to investigate whether chronic exposure to UV-light might also lead to *MYC* copy number changes, 51 AFX and 24 PDS samples were retrospectively analyzed for *MYC* amplification by fluorescence in situ hybridization using a *MYC* and a CEP8 gene probe. Of the 44 analyzable AFX samples, one case showed *MYC* amplification (defined as a *MYC*/CEP8 ratio ≥2.0), whereas 13 cases demonstrated low level copy number gains (defined as *MYC*/CEP8 ratio ≥ 1.2−< 2.0). *MYC* amplification was seen in an AFX sample of extraordinary tumor thickness of 17.5 mm (vs. median 3.25 mm for all samples). Of the 24 PDS cases, five specimen demonstrated *MYC* low level copy number gains. Immunohistochemically, neither the AFX nor the PDS cases showed MYC protein expression.

In summary, these findings rule out that *MYC* amplification is a major genetic driver in the process of AFX or PDS tumorigenesis. However, *MYC* amplification may occur as a late event during AFX development and hence might only be detectable in advanced, thick lesions.

## INTRODUCTION

Atypical fibroxanthoma (AFX), and pleomorphic dermal sarcoma are both rare tumors that typically occur in UV-damaged skin of the elderly. Approximately 90% develop on face, scalp, ears, or neck, with a predominance of the male gender. The remaining ~10% arise on the extremities and trunk [1]. In contrast to AFX, which generally does not recur after complete excision, PDS locally reoccurs in up to 50% and metastasizes in up to 20% [2, 3]. Clinically, lesions usually suggest malignancy because they develop rapidly (over just a few weeks or months) and present as a solitary reddish pink plaque or nodule with central ulceration.

Histologically, AFX tumor cells demonstrate moderate to severe pleomorphism with spindle, epithelioid, or multinucleated forms and atypical mitotic figures. A differentiation from pleomorphic dermal sarcoma (PDS, formerly also classified as cutaneous undifferentiated pleomorphic sarcoma (UPS) or cutaneous malignant fibrous histiocytoma (MFH)) can be difficult. Helbig and colleagues compared oncogene pathways in well-defined AFX and PDS and could demonstrate that AFX and PDS present with similar oncogene expression profiles, such as PT53, CCND1, and CDK4 overexpression [2]. The authors therefore suggested that AFX is the non-infiltrating precursor lesion of PDS, which usually infiltrates into the subcutis [2].

Mild to severe solar elastosis in the AFX/PDS-adjacent normal skin is frequently present underlining the role of UV-radiation for AFX/PDS tumorigenesis. Sun exposure and radiation therapy are known risk factors for developing AFX/PDS. Early publications showed a high rate of UV-induced mutations of the *TP53* gene in AFX (75%; *n* = 8) [4]. Using next generation sequencing, Helbig *et al*. could demonstrate TP53 mutations in 100% (*n* = 5) of PDS and 20% (*n* = 5) of AFX [2]. Recent findings from our own institute revealed that amplification of *MYC* (alias *c-Myc*) occurs in radiation-induced (secondary) angiosarcoma, but not in primary angiosarcoma [5]. *MYC* amplifications are supposed to induce tumorigeneses by increasing genomic instability [6, 7]. Since AFX and PDS primarily occur on UV-light exposed locations, it seemed reasonable to study their *MYC* status.

Recent genetic studies have identified similarities between AFX and PDS, such as deletions of chromosome arms 9p and 13q [8].

In contrast, other groups demonstrated distinct differences between the two entities. Sakamoto and colleagues could show that *HRAS* and *KRAS* mutations are only present in AFX, but not in PDS [9]. Mihic-Probst and colleagues used comparative genomic hybridization to demonstrate distinct chromosomal differences between AFX and PDS, such as losses of 1q, 3p, 5q, 11p, 11q and gains of 5p, 7q, 11q, 12q in PDS but not in AFX [10]. *MYC* is located on chromosome arm 8q. However, information about chromosome 8 alterations in AFX is sparse and fluorescence *in situ* hybridization (FISH) data for *MYC* (8q24) has not been reported so far. Therefore, the present study was undertaken to examine *MYC* in AFX and PDS.

## RESULTS

### Clinical features

A total of 51 AFX patients were included (Table 1). Patients’ age ranged from 67 to 95 years, with a median of 80 years. A predominance of the male gender (39/51 patients, 76%) was observed (Table 1). One of the patients had a recurrence of the tumor at the site of excision after one year although the tumor was resected with a safety margin of 1 cm. Development of metastases and multiple AFX were not observed.

**Table 1:** Clinical characteristics of patients with atypical fibroxanthoma

Patient ID	Age at diagnosis	Gender	Localization	Invasion depth (mm)	Ratio *MYC*/CEN8	Result
1	95	M	Scalp	6.25	1.13	diploid
2	73	M	Scalp	6.5	1.13	diploid
3	71	M	Scalp	3.25	1.33	low level gain
4	87	M	Hand	4.0	1.11	diploid
5	68	M	Scalp	3.75	1.08	diploid
6	72	M	Ear	6.0	1.34	low level gain
7	81	M	Ear	6.25	1.32	low level gain
8	88	M	Scalp	17.5	2.06	amplification
9	67	M	Scalp	7.5	1.14	diploid
10	89	F	Nose	6.0	1.36	low level gain
11	72	M	Scalp	N/A	1.13	diploid
12	80	M	Scalp	N/A	1.12	diploid
13	82	M	Forehead	5.5	1.25	low level gain
14	76	M	Scalp	5.5	1.13	diploid
15	84	M	Ear	5.25	1.28	low level gain
16	68	F	Forehead	2.5	1.13	diploid
17	80	M	Ear	3.0	1.67	low level gain
18	86	M	Scalp	9.5	1.23	low level gain
19	83	M	Scalp	5.75	1.33	low level gain
20	75	M	Cheek	2.0	1.08	diploid
21	80	M	Scalp	8.0	1.07	diploid
22	76	M	Scalp	2.75	1.11	diploid
23	78	M	Scalp	2.0	1.09	diploid
24	79	M	Forehead	2.25	1.11	diploid
25	72	F	Forehead	2.0	1.13	diploid
26	77	M	Scalp	12.0	N/A	N/A
27	87	M	Forehead	N/A	1.11	diploid
28	75	M	Forehead	N/A	1.17	diploid
29	80	M	Forehead	2.0	1.12	diploid
30	80	F	Eyebrow	2.2	1.37	low level gain
31	79	M	Scalp	4.1	1.49	low level gain
32	81	M	Forehead	2.2	N/A	N/A
33	86	F	Cheek	3.5	1.14	diploid
34	76	M	Eyelid	4.15	1.14	diploid
35	76	M	Forehead	10.0	1.13	diploid
36	83	F	Forehead	1.45	1.13	diploid
37	74	F	Forehead	3.0	N/A	N/A
38	87	M	Cheek	N/A	1.11	diploid
39	75	F	Forehead	1.7	1.14	diploid
40	90	F	Eyebrow	2.0	1.13	diploid
41	70	M	Neck	3.2	1.12	diploid
42	85	M	Scalp	2.5	1.13	diploid
43	88	M	Scalp	N/A	N/A	N/A
44	88	M	Scalp	N/A	1.14	diploid
45	84	M	Neck	N/A	N/A	N/A
46	70	F	Forehead	3.0	1.32	low level gain
47	86	F	Cheek	2.0	1.14	diploid
48	85	F	Forehead	1.6	1.13	diploid
49	87	M	Scalp	N/A	1.35	low level gain
50	81	M	Scalp	1.6	N/A	N/A
51	73	M	Scalp	N/A	N/A	N/A

Abbreviations: M: male, F: female, N/A: data not available, light grey: low level amplification, dark grey: amplification.

The single case with *MYC* amplification (*MYC*/CEP8 ratio ≥2.0) is highlighted in dark grey, the 13 cases with low level *MYC* copy number gain (*MYC*/CEN 8 ratio ≥1.2–<2.0) are accentuated in light grey.

Additionally, a cohort of 24 PDS patients was investigated (Table 2). PDS patients´ age ranged from 58 to 92, with a median of 77 years. Similar to the AFX patients, the PDS patients also demonstrated a predominance of the male gender (19/24 patients, 79%) (Table 2). Tumor thickness of the PDS cohort was not available.

**Table 2:** Clinical characteristics of patients with pleomorphic dermal sarcoma

Patient ID	Age at diagnosis	Gender	Localization	Invasion depth (mm)	Ratio *MYC*/CEN8	Result
1	68	M	Scalp	N/A	1.13	diploid
2	89	M	Scalp	N/A	1.11	diploid
3	66	M	Scalp	N/A	1.08	diploid
4	81	M	Scalp	N/A	1.11	diploid
5	58	M	Forehead	N/A	1.08	diploid
6	92	M	Cheek	N/A	1.32	low level gain
7	73	F	Nose	N/A	1.14	diploid
8	74	M	Scalp	N/A	1.13	diploid
9	80	F	Cheek	N/A	1.14	diploid
10	89	M	Forehead	N/A	1.33	low level gain
11	81	F	Cheek	N/A	1.13	diploid
12	88	M	Forehead	N/A	1.12	diploid
13	74	F	Scalp	N/A	1.11	diploid
14	74	M	Shoulder	N/A	1.13	diploid
15	77	M	Scalp	N/A	1.28	low level gain
16	74	M	Scalp	N/A	1.13	diploid
17	71	M	Scalp	N/A	1.13	diploid
18	75	M	Scalp	N/A	1.23	low level gain
19	82	M	Scalp	N/A	1.49	low level gain
20	84	M	Scalp	N/A	1.08	diploid
21	86	M	Scalp	N/A	1.07	diploid
22	86	M	Scalp	N/A	1.11	diploid
23	76	M	Scalp	N/A	1.15	diploid
24	59	F	Shoulder	N/A	1.11	diploid

Abbreviations: M: male, F: female, N/A: data not available, light grey: low level amplification.

The five cases with low level *MYC* copy number gain (*MYC*/CEN 8 ratio ≥1.2–<2.0) are accentuated in light grey.

### Immunohistochemistry

Immunohistochemically, none of the investigated 51 AFX and 24 PDS specimen stained positive for MYC protein expression (data not shown).

### Fluorescence *in situ* hybridization

Interphase FISH analysis for *MYC* was successfully performed on 44/51 AFX and 24/24 PDS cases. Seven AFX cases were not evaluable due to missing cores on the FISH TMA slide.

**Figure 1:**
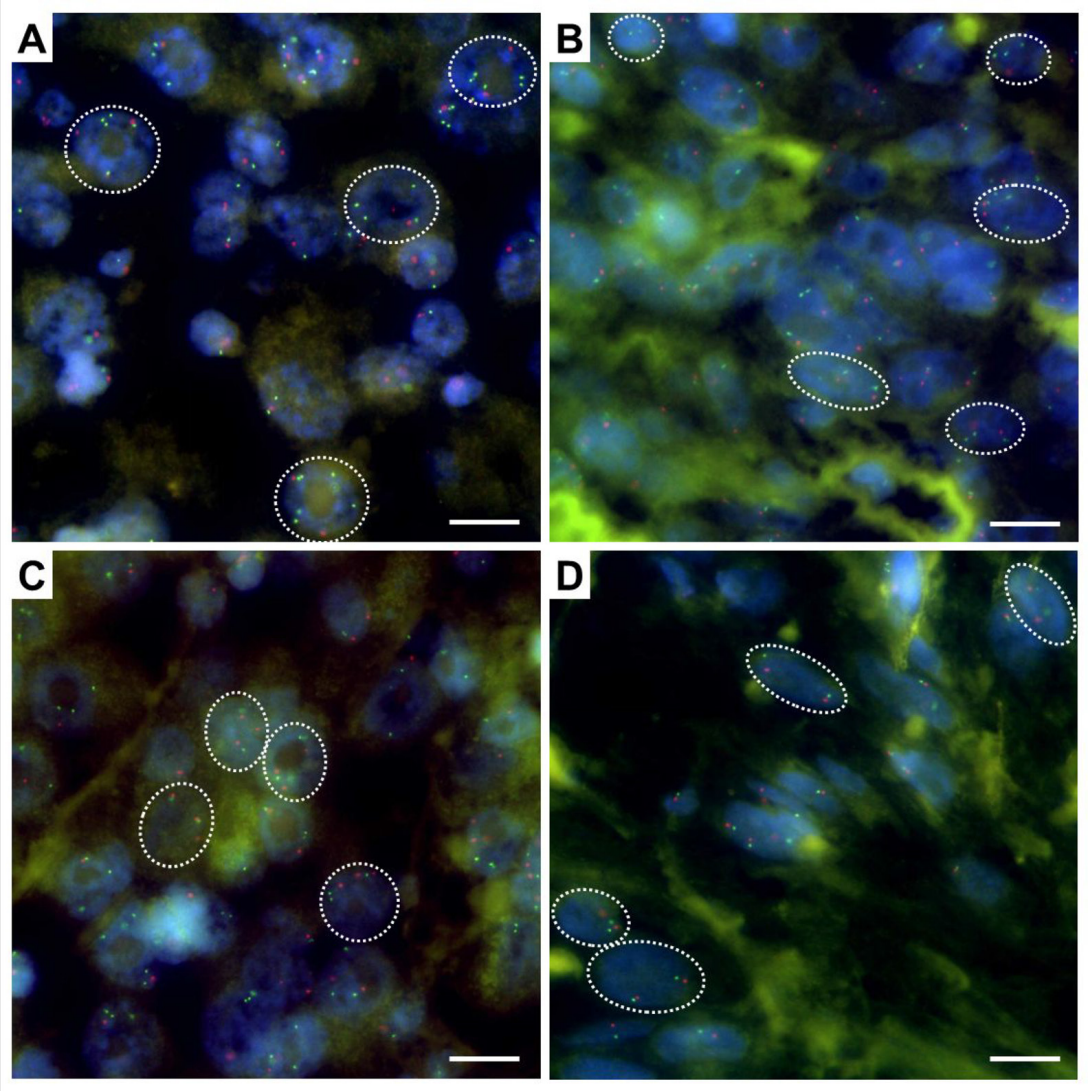
*MYC*/CEN 8 fluorescence in situ hybridization on interphase nulcei in AFX Shown are representative immunofluorescence images of (**A**) *MYC* amplified case (patient ID 8, *MYC*/CEN 8 ratio 2.06), (**B**) case with a low level copy number gain of *MYC* (patient ID 6, *MYC*/CEN 8 ratio 1.34), (**C**) near-polysomic case (patient ID 17, *MYC*/CEN 8 ratio 1.67, on average 2.85 CEN 8 signals per cell), and (**D**) diploid case (patient ID 12, *MYC*/CEN 8 ratio 1.12). Scale bar, 10 μm. Green, *MYC* (8q24) signals; red, centromeric region of chromosome 8.

Interphase FISH analysis on TMA spots from each patient revealed *MYC* amplification (*MYC*/CEP8 ratio 2.06, *MYC* signal number 4.02) in only one AFX case (1/44 = 2.3%) (Figure 1). In 13 other AFX specimens and 5 PDS cases, low level *MYC* gain was detected (13/44 = 29.5% and 5/24 = 21%). The remaining 30 AFX cases (30/45 = 68.2%) and 19 PDS cases (19/24 = 79%) were found to be diploid for *MYC* by FISH analysis (Tables 1 and 2, Supplementary Tables 1 and 2). According to our defined criteria (Material and Methods) no polysomy was observed, however one AFX case and one PDS case fell short with an average of 2.85 and 2.8 CEP8 signals per cell (Supplementary Tables 1 and 2).

**Figure 2:**
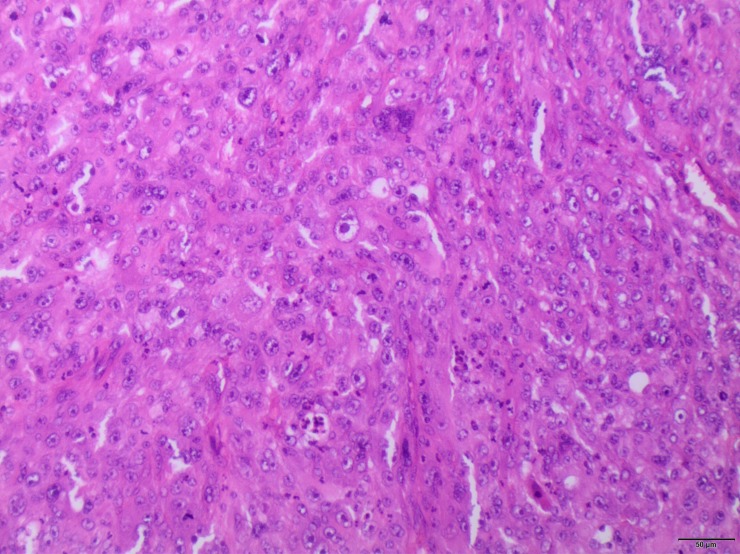
Histopathological examination showing distinct features of AFX, such as pronounced cell pleomorphism with spindle, epithelioid, and multinucleated forms Numerous atypical mitotic figures were present (HE, scale bar 50 μm).

None of the evaluated clinicopathological parameters (tumor invasion thickness, preexisting diseases/malignancies, smoking behavior, location, age, gender) were associated with *MYC* amplification (data not shown). However, it is remarkable that the only *MYC* amplified AFX case showed the highest tumor thickness (17.5 mm) of all cases (17.5 mm vs. median 3.25 mm, range 1.45 to 17.5) (Figure 2).

## DISCUSSION

To the best of our knowledge *MYC* copy number alterations (CNA) have not been studied systematically in AFX or PDS. Our group could link *MYC* amplification to antecedent radiation treatment in angiosarcoma [5] and various other types of radiation treated sarcoma [6]. Besides, FISH analysis demonstrated that malignant melanoma, a tumor that can also be induced by UV-radiation, shows *MYC* copy number gain [11]. It was therefore reasonable to postulate that chronic sun exposure, the major risk factor for developing AFX or PDS, might also lead to *MYC* CNA. Like radiotherapy, UV-radiation can cause direct (UVB) and indirect (UVA) DNA damage [12] and plays an important role in skin cancer development [4, 13]. While UVA radiation usually causes single-strand breaks in DNA, UVB radiation can lead to double-strand DNA breakage [12]. We found that *MYC* amplification is a rare event in AFX and PDS; 13 of 44 (29.5%) AFX specimen showed *MYC* low level copy number gain and only one AFX patient (2.3%) had a *MYC* amplification. None of the PDS cases showed a *MYC* amplification and only 5 of 24 (21%) cases demonstrated *MYC* low level copy number gain. The one AFX case with a *MYC* amplification was an 88-year old male patient with an advanced AFX (17.5 mm thickness, ulcerated). However, these results are not unexpected since chronic UV radiation has a much lower energy (UVA to UVB: 3.26 to 4.43 eV) than the dosages frequently used for radiotherapy (70 Gray in a 70 kg patient: ~3.06 × 10^21^ eV).

The amplification of *MYC* did not have an impact on tumor cell morphology, a fact already known from angiosarcoma where *MYC* amplification did not correlate with the histological grade or other morphologic features [5]. A prognostic role for *MYC* could not be demonstrated since only one case in this study showed local recurrence and none of the cases presented with metastases during follow-up.

Based on the detected low frequency of *MYC* amplification in AFX and PDS it is difficult to speculate about its role for AFX/PDS tumorigenesis. Apparently it is a rare genetic event and not specific for these entities. Furthermore, sun exposure does not seem to lead to the same genetic alterations that are known from radiation induced angiosarcoma, in which *MYC* amplification was present in 55% of the cases [5]. Besides of secondary angiosarcoma [5], *MYC* amplification has also been reported in malignant fibrous histiocytoma [14], high grade chondrosarcoma [15], epitheloid sarcoma of the proximal type [16], and in high grade myxoid liposarcomas [17]. For leiomyosarcoma, *MYC* amplification was shown to have an adverse prognostic impact [18]. In total, it seems that in certain sarcoma entities *MYC* amplification is associated with higher tumor grades and worse prognosis. *MYC* CNA in high grade adult soft tissue sarcomas have been reported as rare events (7 of 207, 3.4%) [19]. In an own study, we observed a *MYC* amplification frequency of 6% for non-radiated PDS (vs. 29% for radiation induced sarcoma) [6]. Helbig and colleagues found amplifications and deletions in 6 of 27 PDS cases but not in AFX [20]. Becerikli and colleagues could not detect any *MYC* CNA in 19 PDS samples [21]. Furthermore, it has been reported that AFX and PDS may share distinct genetic events (e.g., 9p & 13q losses) [8]. Thus, after acquiring additional genetic alteration, AFX could progress into PDS and may therefore present a superficial form of PDS. However, our findings do rule out that *MYC* amplification is a major genetic driver for the development of AFX or PDS since it was a very rare event, the only AFX case with *MYC* amplification did not show histological signs for transition into PDS, even though it was the most advanced lesion and none of the PDS cases demonstrated a *MYC* amplification. Immunhistochemically, none of the investigated cases was positive for MYC protein expression. Apparently, even in the single amplified AFX case, the level of *MYC* amplification was not sufficient to result into immunohistochemically detectable MYC protein expression. It still has to be determined by which pathway chronic sun exposure leads to the formation of AFX/PDS, most certainly *MYC* amplification can be excluded as the major genetic driver. However, our data indicate that *MYC* amplification occurs as a late event during the tumorigenesis and might only be detectable in advanced AFX lesions.

## MATERIALS AND METHODS

### Patients and tissue microarray

In total, 51 AFX formalin-fixed paraffin-embedded (FFPE) tumor specimens were retrospectively collected from the archive of the Institute of Pathology and Dermatohistological Laboratory Prof. Kind. The 24 PDS FFPE specimens were provided from the archive of the Institute for Pathology of the University Hospital Cologne. Additionally, demographic and clinical characteristics, such as age, gender, location, tumor specific data, in some cases co-morbidities and smoking behavior, were collected. All lesions were reassessed by two independent pathologists (TG, AQ) to reassure the AFX/PDS diagnosis. The study was approved by the local board of ethics of the University Medical Center Mannheim (2014-835R-MA) and the University Hospital Cologne (Registration No. 15-307).

Tumor areas were marked on whole sections and each tumor sample was assembled on tissue microarrays (TMA) in triplicates (Mannheim TMA 1) and duplicates (Cologne TMA 1 and Mannheim TMA 2) using TissueMax Automated and Personal Tissue Microarrayers according to the manufacturer's protocol [22].

### Immunohistochemistry

Tissue sections were stained for MYC (clone N262, Santa Cruz, 1:50). Sections were subjected to heat-induced EDTA-based antigen retrieval. Antibody binding was visualized using the EnVision Detection System, Peroxidase/DAB, Rabbit/Mouse (cat# K5007, Dako) according to the manufacturer's instructions.

### Fluorescence *in situ* hybridization

FISH for quantitation of *MYC* and chromosome 8 centromere (CEP8) was performed on 5 μm FFPE tissue sections using the ZytoLight SPEC *MYC*/CEN 8 Dual Color Probe (Zytomed Systems GmbH, Berlin, Germany). Deparaffinization, denaturation and hybridization were done according to the manufacturer's instructions. For denaturation and hybridization a StatSpin Hybridizer instrument (cat # S2450, Dako, Hamburg, Germany) was used. Hybridized slides were counterstained with Vectashield Antifade Mounting Medium with DAPI (H-1200, Vector Laboratories, Inc., Burlingame, CA, USA) and then analyzed using an Olympus BX41 fluorescence microscope (Olympus Deutschland GmbH, Hamburg, Germany) with optical filters for DAPI, SpectrumGreen and SpectrumOrange (Olympus) with a UPlanSApo 60x objective (oil, numerical aperture 1.35; Olympus). The microscope was connected to an F-View II CCD-Camera (Soft Imaging System GmbH, Muenster, Germany). Cell^F software (Olympus Soft Imaging Solutions GmbH, Muenster, Germany) was used to acquire representative images with each filter. Sixty interphase nuclei were counted per sample. Following previous publications [23–25] the following signal patterns were defined:

- *MYC* amplification: *MYC*/CEP8 ratio ≥2.0
- *MYC* low level gain: *MYC*/CEP8 ratio ≥1.2–<2.0
- Polysomy: CEP8 (≥3.0)

## SUPPLEMENTARY MATERIALS AND TABLES




