# Supplementary material for: MYC gene amplification is a rare event in atypical fibroxanthoma and pleomorphic dermal sarcoma
Source: Oncotarget. 2018 Apr 20;9(30):21182–9. doi: 10.18632/oncotarget.24997 (PMC5940384; doi:10.18632/oncotarget.24997)
Supplement: Supplementary file 1 [file oncotarget-09-21182-s001.pdf]

MYC gene amplification is a rare event in atypical fibroxanthoma and pleomorphic dermal sarcoma

SUPPLEMENTARY MATERIALS

Supplementary Table 1: *MYC* FISH results for the 51 atypical fibroxanthomas. See Supplementary_Table_1

Supplementary Table 2: FISH results of patients with pleomorphic dermal sarcoma

TMA	Patient ID	Number of counted nuclei	Average *MYC* signal number	Average CEP8 signal number	Ratio *MYC*/CEP8	Result	Number of tissue cores with tumor
1	1	60	2.03	1.79	1.13	diploid	2
1	2	60	2.14	1.93	1.11	diploid	2
1	3	60	2.20	2.13	1.08	diploid	2
1	4	60	2.10	1.90	1.11	diploid	2
1	5	60	2.30	2.13	1.08	diploid	2
1	6	60	2.33	1.77	1.32	low level gain	2
1	7	60	2.13	1.87	1.14	diploid	2
1	8	60	2.07	1.83	1.13	diploid	2
1	9	60	2.24	1.97	1.14	diploid	2
1	10	60	2.52	1.90	1.33	low level gain	2
1	11	60	2.07	1.83	1.13	diploid	2
1	12	60	2.17	1.93	1.12	diploid	2
1	13	60	2.14	1.93	1.11	diploid	2
1	14	60	2.03	1.79	1.13	diploid	2
1	15	60	2.73	2.13	1.28	low level gain	2
1	16	60	2.03	1.79	1.13	diploid	2
1	17	60	2.03	2.80	1.13	diploid	2
1	18	60	2.63	2.14	1.23	low level gain	2
1	19	60	2.5	1.68	1.49	low level gain	2
1	20	60	2.00	1.86	1.08	diploid	2
1	21	60	2.10	1.97	1.07	diploid	2
1	22	60	2.14	1.93	1.11	diploid	2
1	23	60	1.98	1.75	1.15	diploid	2
1	24	60	2.07	1.86	1.11	diploid	2

The single case with *MYC* amplification (*MYC*/CEP8 ratio ≥2.0) is highlighted in dark grey, the 13 cases with low level *MYC* copy number gain (*MYC*/CEN 8 ratio ≥1.2 – <2.0) are accentuated in light grey.
