# Supplementary material for: MYC gene amplification is a rare event in atypical fibroxanthoma and pleomorphic dermal sarcoma
Source: Oncotarget. 2018 Apr 20;9(30):21182–9. doi: 10.18632/oncotarget.24997 (PMC5940384; doi:10.18632/oncotarget.24997)
Supplement: Supplementary file 2 [file oncotarget-09-21182-s002.pdf]

Supplementary Table 1. *MYC* FISH results for the 51 atypical fibroxanthomas.

TMA	Patient ID	Number of counted nuclei	Average *MYC* signal number	Average CEP8 signal number	Ratio *MYC*/CEP8	Result	Number of tissue cores with tumor
1	1	60	2.07	1.83	1.13	diploid	2
1	2	60	2.10	1.86	1.13	diploid	3
1	3	60	2.52	1.90	1.33	low level gain	3
1	4	60	2.10	1.90	1.11	diploid	3
1	5	60	2.30	2.13	1.08	diploid	3
1	6	60	2.63	1.96	1.34	low level gain	1
1	7	60	2.33	1.77	1.32	low level gain	3
1	8	60	4.02	1.95	2.06	amplification	3
1	9	60	2.24	1.97	1.14	diploid	3
1	10	60	2.35	1.73	1.36	low level gain	2
1	11	60	2.07	1.83	1.13	diploid	3
1	12	60	2.17	1.93	1.12	diploid	3
1	13	60	2.82	2.25	1.25	low level gain	3
1	14	60	2.03	1.79	1.13	diploid	2
1	15	60	2.73	2.13	1.28	low level gain	2
1	16	60	2.03	1.79	1.13	diploid	1
1	17	20	4.75	2.85	1.67	low level gain	1
1	18	35	2.63	2.14	1.23	low level gain	1
1	19	60	2.34	1.76	1.33	low level gain	2
1	20	60	2.00	1.86	1.08	diploid	2
1	21	60	2.10	1.97	1.07	diploid	2
1	22	60	2.14	1.93	1.11	diploid	2
1	23	60	2.07	1.90	1.09	diploid	2
1	24	60	2.07	1.86	1.11	diploid	2
2	25	60	2.03	1.80	1.13	diploid	2
2	26	N/A	N/A	N/A	N/A	N/A	0
2	27	20	2.00	1.80	1.11	diploid	1
2	28	60	2.13	1.82	1.17	diploid	2
2	29	60	2.13	1.90	1.12	diploid	2
2	30	60	2.35	1.72	1.37	low level gain	2
2	31	60	2.50	1.68	1.49	low level gain	2
2	32	N/A	N/A	N/A	N/A	N/A	0
2	33	60	2.13	1.87	1.14	diploid	2
2	34	60	2.17	1.90	1.14	diploid	2
2	35	60	2.07	1.83	1.13	diploid	2
2	36	60	2.00	1.77	1.13	diploid	1
2	37	N/A	N/A	N/A	N/A	N/A	0
2	38	20	2.00	1.80	1.11	diploid	1
2	39	60	1.97	1.73	1.14	diploid	1
2	40	60	2.03	1.80	1.13	diploid	1
2	41	60	2.10	1.87	1.12	diploid	1

2	42	60	2.07	1.83	1.13	diploid	2
2	43	N/A	N/A	N/A	N/A	N/A	0
2	44	60	2.13	1.87	1.14	diploid	2
2	45	N/A	N/A	N/A	N/A	N/A	0
2	46	60	2.37	1.80	1.32	low level gain	1
2	47	40	2.05	1.80	1.14	diploid	1
2	48	60	2.07	1.83	1.13	diploid	1
2	49	60	2.30	1.70	1.35	low level gain	2
2	50	N/A	N/A	N/A	N/A	N/A	0
2	51	N/A	N/A	N/A	N/A	N/A	0

The single case with *MYC* amplification (*MYC*/CEP8 ratio ≥2.0) is highlighted in dark grey, the 13 cases with low level *MYC* copy number gain (*MYC*/CEN 8 ratio ≥1.2 – <2.0) are accentuated in light grey.

N/A: data not available, light grey: low level amplification, dark grey: amplification
